# Plasmablasts as Migratory IgG-Producing Cells in the Pathogenesis of Neuromyelitis Optica

**DOI:** 10.1371/journal.pone.0083036

**Published:** 2013-12-10

**Authors:** Norio Chihara, Toshimasa Aranami, Shinji Oki, Takako Matsuoka, Masakazu Nakamura, Hitaru Kishida, Kazumasa Yokoyama, Yoshiyuki Kuroiwa, Nobutaka Hattori, Tomoko Okamoto, Miho Murata, Tatsushi Toda, Sachiko Miyake, Takashi Yamamura

**Affiliations:** 1 Department of Immunology, National Institute of Neuroscience, National Center of Neurology and Psychiatry (NCNP), Tokyo, Japan; 2 Department of Neurology, Kobe University Graduate School of Medicine, Kobe, Japan; 3 Department of Neurology, Yokohama City University Graduate School of Medicine, Yokohama, Japan; 4 Department of Neurology, Juntendo University Graduate School of Medicine, Tokyo, Japan; 5 Department of Neurology, National Center Hospital, NCNP, Tokyo, Japan; 6 Multiple Sclerosis Center, National Center Hospital, NCNP, Tokyo, Japan; Innsbruck Medical University, Austria

## Abstract

Neuromyelitis optica (NMO) is an inflammatory disease characterized by recurrent attacks of optic neuritis and myelitis. It is generally accepted that autoantibodies against aquaporin 4 water channel protein play a pathogenic role in neuromyelitis optica. We have recently reported that plasmablasts are increased in the peripheral blood of this autoimmune disease, and are capable of producing autoantibodies against aquaporin 4. Here, we demonstrate that CD138^+^HLA-DR^+^ plasmablasts, a subset of IgG-producing cells, are increased in the peripheral blood and are enriched among the cerebrospinal fluid (CSF) lymphocytes during the relapse of neuromyelitis optica. Notably, these CD138^+^HLA-DR^+^ plasmablasts overexpress CXCR3, whose ligands are present in the cerebrospinal fluid during the relapse of neuromyelitis optica. These results led us to speculate that plasmablasts producing anti-aquaporin 4 autoantibodies might traffic toward the central nervous system (CNS). Furthermore, we performed single-cell sorting of plasmablasts from peripheral blood and CSF samples from NMO and sequenced the complementarity-determining regions (CDRs) of the IgG heavy chain expressed by the sorted plasmablast clones. There were high frequencies of mutations in the CDRs compared with framework regions, indicating that these plasmablast clones would represent a post-germinal center B-cell lineage. Consistent with the preceding results, the plasmablast clones from the peripheral blood shared the same CDR sequences with the clones from the CSF. These results indicate that IgG-producing plasmablasts, which are guided by helper T-cells, may migrate from the peripheral blood preferentially to the CSF. Since migratory plasmablasts could be involved in the inflammatory pathology of NMO, the B-cell subset and their migration might be an attractive therapeutic target.

## Introduction

Neuromyelitis optica (NMO) is a rare inflammatory disease primarily affecting the optic nerve and spinal cord, with relatively sparing brain white matter [1]. NMO exhibits a relapsing–remitting course reminiscent of multiple sclerosis (MS) and was previously thought to be a variant of MS. However, NMO is now considered to have a unique pathogenesis characterized by the elevation of autoantibodies against aquaporin 4 (AQP4) [2,3]. NMO is more often accompanied by the elevation of serum autoantibodies including anti-nuclear, anti-SSA, and anti-SSB antibodies than MS. Notably, the relapses of NMO are not prevented but rather triggered by disease-modifying agents prescribed for MS, including interferon-beta[4,5]. Recent studies have indicated that primary autoimmune targets in NMO can be astrocytes, which abundantly express AQP4 in the end foot processes [6–8]. Consistently, inflammatory lesions of NMO are surrounded by deposits of antibodies and complement that are associated with necrotic astrocytes, whereas AQP4 expression in astrocytes is downregulated in the early stage of NMO [6,7]. Moreover, large amounts of glial fibrillary acidic protein (GFAP) can be detected in the cerebrospinal fluid (CSF) of NMO patients during relapse [8]. Experimentally, systemic injection of large quantities of anti-AQP4 autoantibodies (AQP4Ab) from patients’ sera exacerbated inflammatory pathology as well as clinical signs of experimental autoimmune encephalomyelitis in rats [9,10]. In this model of central nervous system (CNS) autoimmunity, the blood-brain barrier (BBB) integrity is disrupted following T-cell–mediated inflammation. In addition, similar astrocyte pathology was evoked in mouse brain by directly injecting AQP4Ab with human complement [11]. However, in human NMO, it remains unclear whether sufficient quantities of AQP4Ab may enter the CNS from the periphery so that they can cause the astrocyte pathology. This leaves room for a significant role of local production of AQP4Ab in the pathogenesis of NMO.

Recently, we reported that plasmablasts (PBs), bearing a phenotype of CD19^int^CD27^high^CD38^high^CD180^-^, are B-cells selectively increased in the peripheral blood of NMO, compared with control subjects [12]. A significant increase in PBs was observed during remission of NMO, but the increase was more remarkable during relapse in individual patients. Moreover, we identified the PBs as AQP4-Ab–producers in the peripheral blood of NMO. In principle, pathogen-activated B-cells migrate to lymphoid organs, and differentiate into PBs or memory B-cells (mB) within a germinal center. Some PBs move to the bone marrow and give rise to long-lived plasma cells, which contribute to maintaining the levels of serum antibodies against pathogens. The other PBs would die after undergoing apoptosis, or survive in lymphoid or non-lymphoid tissues in the inflammatory milieus [13]. However, the fate of the circulating PBs in the peripheral blood of NMO remains unclear. 

The CSF of NMO patients reportedly contains much lower titers of AQP4-Ab than their peripheral blood [14], which is also supported by our unpublished results. On the other hand, cytokines preferring B-cell activation and survival, such as interleukin (IL)-6 or B-cell activating factor (BAFF), are elevated in the CSF of NMO patients [15,16]. Thus, low titers of AQP4-Ab in the CSF would not exclude the possibility of intrathecal AQP4-Ab production, but could reflect its deposition in inflammatory lesions. In this respect, the presence of AQP4-Ab–producing B-cells in the CSF was demonstrated in a patient with NMO [10], although the origin and identity of the cells were not fully characterized. 

Here, we report that CSF lymphocytes obtained during the relapse of NMO are enriched in a subpopulation of PBs expressing CD138 and human leukocyte antigen (HLA)-DR (CD138^+^HLA-DR^+^ PB). These cells correspond to recently activated PBs, and also increase in the peripheral blood of patients with NMO during relapse. We found that during the relapse, the activated PB cells in the peripheral blood selectively upregulated CXCR3, a receptor for CXCL10 (interferon gamma-induced protein 10, IP-10) elevated in the CSF of NMO [17]. In contrast, PB expression of CXCR4, a receptor for bone marrow trafficking, was not altered during relapse. In parallel, we examined PB phenotypes in recently vaccinated healthy subjects. In these subjects, activated PBs increased significantly in number but did not upregulate either CXCR3 or CXCR4. These results led us to suspect that the upregulation of CXCR3 during the relapse of NMO might confer the PBs the ability to migrate to the CNS. Moreover, single-cell analysis of the antibodies variable region sequences revealed that the same complementarity-determining region (CDR) sequences in the γ chains were detected in PB clones derived from peripheral blood and from CSF. These results indicate that the CXCR3^+^-activated PBs in the peripheral blood preferentially migrate to the CSF during the relapse of NMO and take part in the formation of the inflammatory pathology. 

## Materials and Methods

### Ethics Statement

Informed consent was obtained from all participants after the nature and possible consequences of the studies were explained. The study was approved by the Ethics Committee of the each participated Institute or University; Ethics Committee of National Center of Neurology and Psychiatry, Ethics Committee for Medical Science of Kobe Graduate School of Medicine, Ethics Committee for Medical Research of Yokohama City University, and Ethics Committee of Juntendo University Hospital. Participants provided their written informed consent to participate in this study.

### Patients and Controls

A cohort of 20 AQP4-Ab-seropositive patients was recruited at the National Center of Neurology and Psychiatry (NCNP) Hospital, Juntendo University Hospital, Yokohama City University Hospital, and Kobe University Hospital. Each patient either met the revised NMO diagnostic criteria [18] or was diagnosed with NMO spectrum disorder (NMOSD) [3]. Serum AQP4-Ab levels were measured with a previously reported cell-based assay using AQP4 transfectants, provided by courtesy of Dr. Kazuo Fujihara at Tohoku University [14]. Eight age-matched patients with MS were enrolled as controls. All MS patients fulfilled the McDonald diagnostic criteria [19]. Clinical demographics of the patients are summarized in Table **1**.

**Table 1:** The clinical profiles of patients with neuromyelitis optica (NMO)/NMO NMO spectrum disorder (NMOSD) and multiple sclerosis (MS).

**Patients**	**NMO/NMOSD**	**MS**
Number	20	8
Age	49.4 ± 3.9	43.0 ± 4.8
Male:Female	4:16	4:4
Disease duration	8.1 ± 2.0	9.9 ± 3.7
Relapses in the last 2 years	1.2 ± 0.3	2.3 ± 0.5
EDSS**^*∗*^** score in disease remission	3.3 ± 0.5	4.1 ± 1.0

The values are expressed as the numbers or means ± standard error of the mean (SEM)

^∗^ Expanded disability status scale

None of the seropositive patients or patients with MS had received intravenous (i.v.) corticosteroids, plasma exchange, or i.v. immunoglobulins for at least one month before blood or CSF sampling. Peripheral blood samples with or without CSF samples were obtained from seven seropositive NMO patients during relapse before they received intensive therapy such as i.v. corticosteroids. There were no significant differences among the NMO patients who provided samples during relapse or during remission, with regard to age, sex, disease duration, 2-year-relapse rate, expanded disability status scale (EDSS), and secondary prevention therapy. Peripheral blood was also taken from six healthy subjects before and one week after annual influenza vaccinations in 2010 to compare their PB cell phenotypes with those from NMO patients. 

### Flow Cytometry Analysis and Cell Sorting

Peripheral blood mononuclear cells (PBMCs) were separated using density centrifugation on Ficoll-Paque PLUS (GE Healthcare Biosciences). CSF cells were directly stained for surface markers after centrifugation. FACSCanto II and FACSCalibur flow cytometers (BD Biosciences) were used for cell analysis along with the FACSAria II (BD Biosciences) cell sorter. We prepared samples with the same concentration of staining antibody and same fluorescence-activated cell sorting (FACS) setting (i.e., background autofluorescence control was set at the same levels and setting compensation was performed for each experiment using single-stained cells as compensation controls). The following monoclonal antibodies (mAbs) were used for flow cytometry analysis and cell sorting: anti-CD19 conjugated with electron-coupled dye (ECD), anit-CD38 conjugated with FITC, anti-HLA-DR conjugated with fluorescein isothiocyanate (FITC), anti-CD3 conjugated with FITC, and anti-CD138 conjugated with phycoerythrin (PE; Beckman Coulter); anti-CD38 conjugated with peridinin chlorophyll-protein complex (PerCP)-Cy5.5, anti-CD138 conjugated with PerCP-Cy5.5, anti-CD27 conjugated with Pacific Blue, anti-CXCR3 conjugated with PerCP-Cy5.5, anti-CXCR4 conjugated with biotin or with PE-Cy7-streptavidin (BioLegend); anti-CD19 conjugated with allophycocyanin (APC)-Cy7, anti-CD27 conjugated with PE-Cy7, anti-CD180 conjugated with PE, and anti-IgG conjugated with FITC (BD Biosciences); anti-CCR10 conjugated with APC; and anti-CXCR3 conjugated with FITC (R&D systems) 

### Sequencing Analysis of Immunoglobulin Gene Variable Regions

Since IgA-secreting PBs reportedly express CCR10 [20], we sorted CCR10^-^PB cells （CD3^−^CD19^int^CD27^high^CD38^high^CD180^−^CCR10^−^）to obtain IgG-secreting PB cells. These PB cells were single-cell sorted into 96-well polymerase chain reaction (PCR) plates containing RNase inhibitor (Promega). We determined the variable region sequences of immunoglobulin genes in the sorted PBs according to a previously described protocol with some minor modifications [21]. Briefly, cDNA fragments of the variable regions of heavy chain (V_H_) and light chain (V_Kappa_) in each sorted cell were amplified by reverse transcription (RT)-PCR. High-fidelity Taq polymerase (Takara Bio) was used to avoid incorrect amplification. The reactions were followed by nested PCR using primer cocktails [21]. V gamma and V kappa cDNA fragments obtained from the first patient were cloned into pMD20-T vectors using Mighty TA-cloning reagents (Takara Bio). More than two clones derived from each single-cell sorted were sequenced to confirm the uniformity of the single PB sorting. Moreover, cDNA fragments of the V gamma genes obtained from another patient were directly sequenced using nested primer sets.

### Data Analysis

Flow cytometry data were analyzed using the FlowJo software (Tomy Digital Biology). Statistical analysis was performed using Prism (GraphPad Software). In addition, Wilcoxon signed rank test, Mann–Whitney U test, or one-way analysis of variance (ANOVA) post-hoc test was used when appropriate. 

## Results

### Predominance of PBs in CSF from NMO during relapse

**Figure 1:**
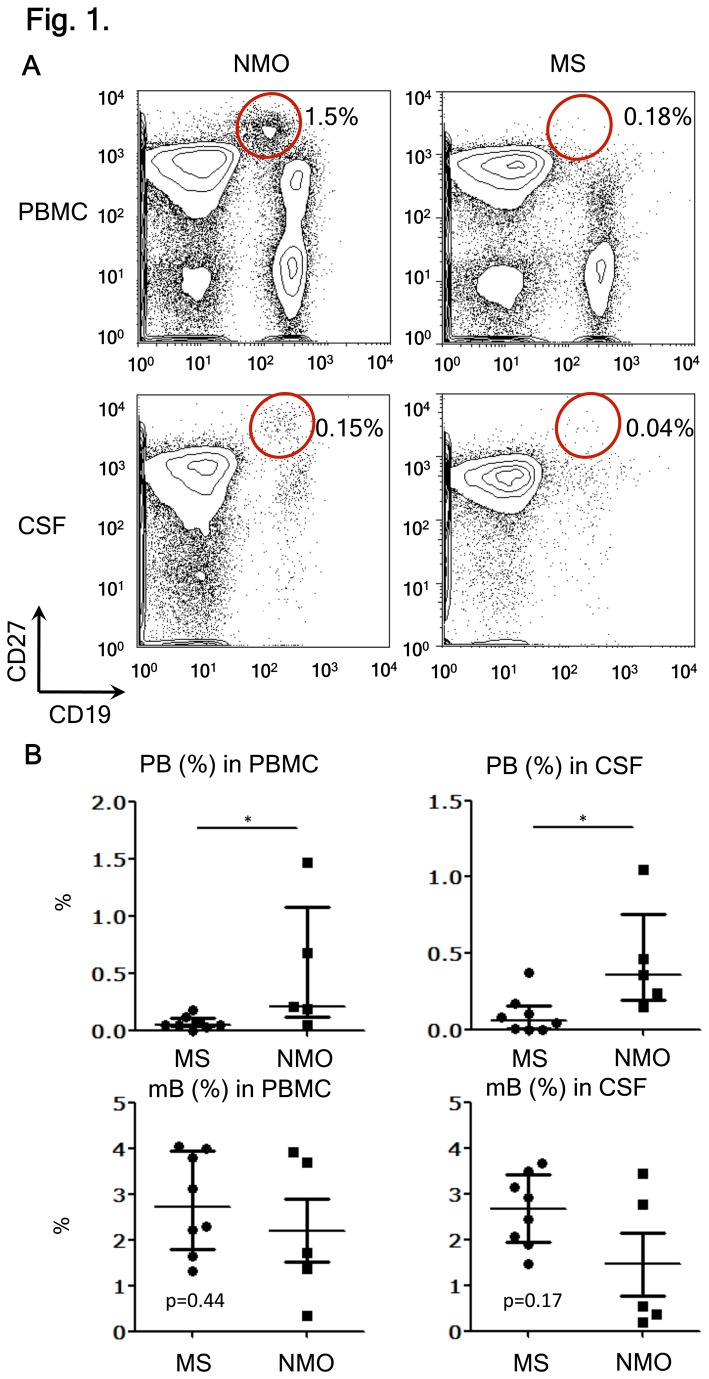
Selective increase of plasmablasts (PBs) during relapse of NMO. (A) B-cell subpopulation analysis by flow cytometry. Peripheral blood mononuclear cell (PBMC) and cerebrospinal fluid (CSF) cells were obtained during relapse of neuromyelitis optica (NMO) or multiple sclerosis (MS) and were stained with fluorescence-conjugated anti-CD19, -CD27, -CD38, and -CD180 monoclonal antibodies (mAbs). PB cells (CD19^int^CD27^high^) were encircled after observing that they also bear the phenotype of CD38^high^CD180^−^ (Figure S1). Values represent the percentages of PB cells among all mononuclear cells. (B) The proportion of PB and memory B-cells (mB) in PBMC and CSF from MS and NMO during relapse. The data were obtained from eight patients with MS and five with NMO [*p < 0.05 by Mann–Whitney test; each error bar represents the median ± interquartile range (IQR)].

We previously showed a significant increase of PB (CD19^int^CD27^high^CD38^high^CD180^-^) in the peripheral blood of NMO patients [12]. However, it remained unclear whether the PBs were also present in the CSF of NMO patients. In an extension study, we analyzed pairs of CSF and peripheral blood from NMO and MS patients. These samples were obtained during disease relapse. We have confirmed that CD27^+^ cells among CD19^+^ B-cells are more frequently found in the CSF (approximately 70%) than in the blood (30%) not only in MS but also in NMO (Figure **1A** and Figure **S1**). CD27 is a marker for mB carrying somatically mutated variable region genes [22]. More interestingly, the proportions of the PB cells were increased in the CSF lymphocytes from NMO compared to those from MS. In contrast, the proportions of mB (CD27^+^CD38^low–mid^CD180^+^ cells) tended to decrease reciprocally, even though it was not statistically significant (Figure **1B**). The proportions of the CD19^+^ B-cells and CD19^+^CD27^+^ B-cells among the PBMC and CSF were not different between NMO and MS patients (Figure **S2**). However, the frequency of PB cells among CD19^+^ B-cells increased significantly in the peripheral blood and CSF of NMO patients compared to that of MS patients (Figure **S3**). An additional study on three NMO patients during remission did not reveal the presence of PBs in the CSF, despite the presence of mB. Therefore, we speculated that the PB cells in the CSF might play an active role during the relapse of NMO. 

### PB cells infiltrating CNS are CD138^+^HLA-DR^+^

**Figure 2:**
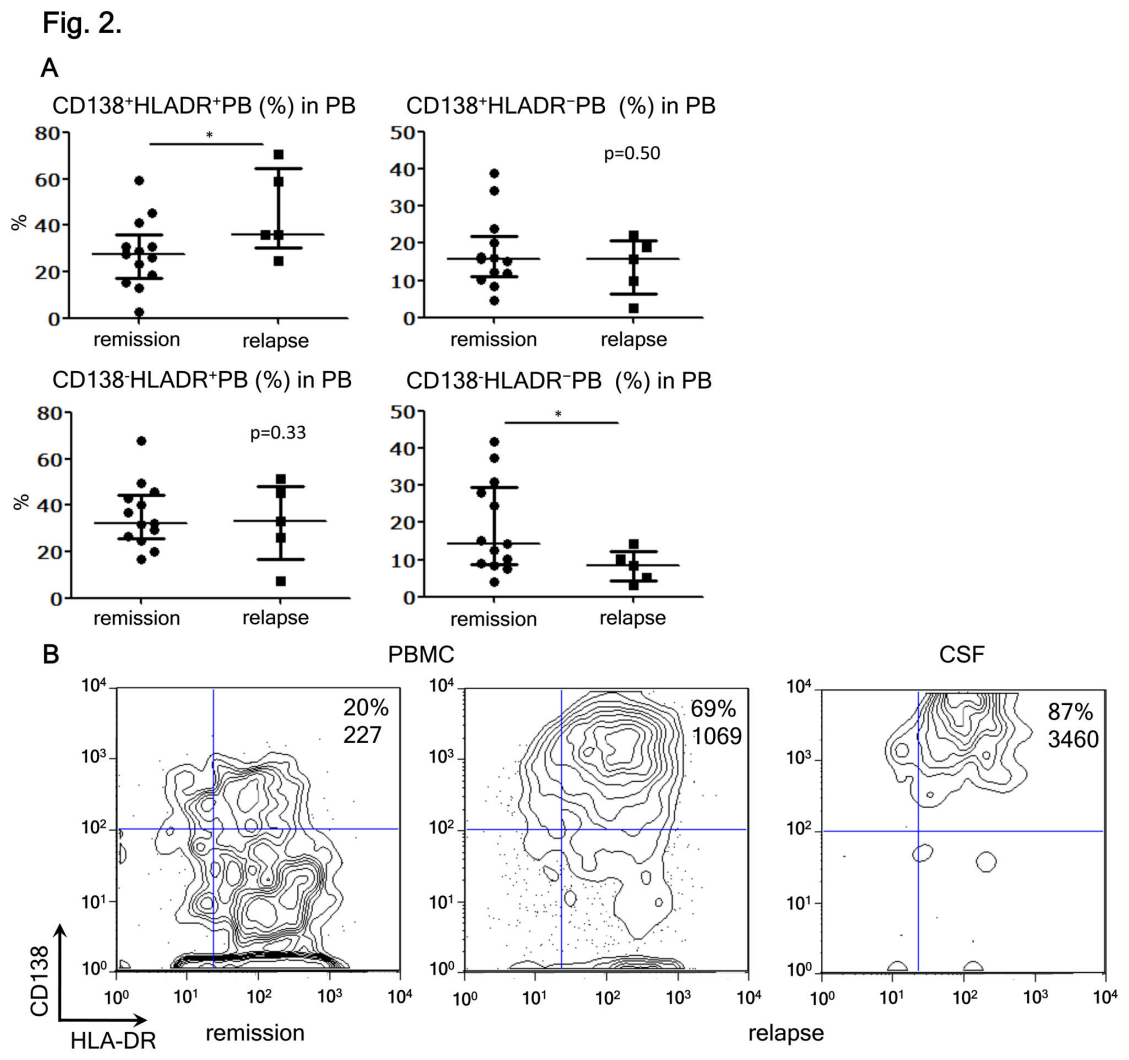
Kinetics of CD138^+^HLA-DR^+^ PB during relapse of NMO. (A) Analysis of pooled peripheral blood mononuclear cells (PBMC) data from neuromyelitis optica (NMO) in remission and relapse. The plasmablasts (PBs) were subdivided into four subpopulations by considering the expression of CD138 and HLA-DR. The individual data show the percentages of each PB subpopulation among the total PB [*p < 0.05 by Mann–Whitney test; each error bar represents the median ± interquartile range (IQR)]. (B) Enrichment of CD138^+^HLA-DR^+^ PBs in the CSF. The PBMC and CSF cells were obtained from NMO during relapse. The values indicate the percentages of CD138^+^HLA-DR^+^ PBs among the total PB. The expression level of CD138 was assessed by mean fluorescence intensity (MFI). Representative data of one out of three different cases are shown.

We asked whether a particular PB subset might exhibit more drastic changes than the whole PB cells. Then, we analyzed the phenotypes of PB subpopulations in the peripheral blood, using antibodies against CD138 and HLA-DR. We showed previously that some PBs in NMO would express the plasma cell marker CD138 [12]. CD138 is known to be an adhesion molecule as well as a negative regulator of cell migration [23]. Both CD138^+^ and CD138^−^ PBs are able to produce AQP4-Ab [12]. However, the mutual relationship of these PB subsets remained obscure. The expression of HLA-DR was examined, because PB cells that produce IgG autoantibodies reportedly express HLA-DR [24]. We found that only the proportion of CD138^+^HLA-DR^+^ PBs significantly increased during the relapse of NMO, compared with remission (Figure **2A**). More importantly, we found that the CD138^+^HLA-DR^+^ PBs comprised the vast majority of PBs in the CSF of NMO patients in relapse (Figure **2B**). Since CD138 is thought to be upregulated in activated cells [23], we speculated that antibody-producing PB cells (CD138^+^HLA-DR^+^ PB) were activated in the periphery in the patients during relapse, and that they might preferentially be recruited to the CSF and be further activated. 

### CD138^+^HLA-DR^+^ PB cells represent recently differentiated IgG-producing PBs

**Figure 3:**
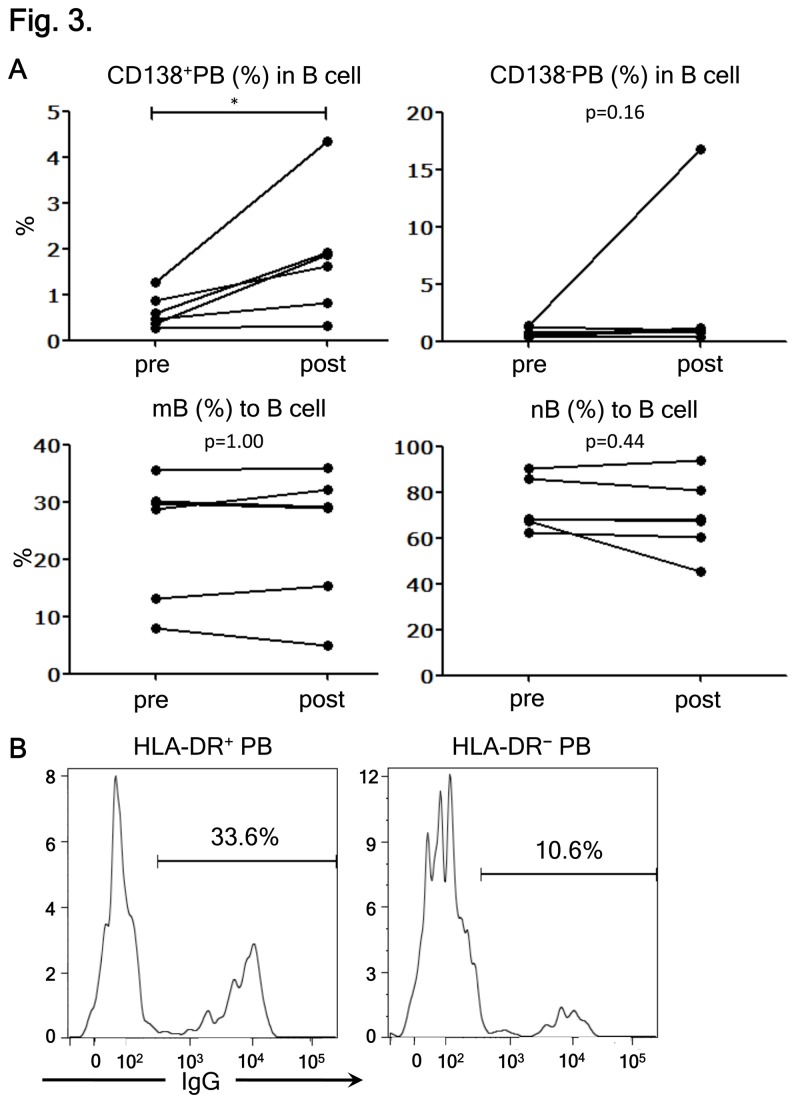
CD138^+^HLA-DR^+^ plasmablasts (PB) cells are recently differentiated IgG-producing PB. (A) The effects of influenza vaccination on the frequencies of B-cell subpopulations were analyzed. The frequency of each B-cell subpopulation derived from the peripheral blood of healthy subjects before (pre) and seven days after vaccination (post) is shown. Each line connects the values obtained from a single subject (*p < 0.05 by Wilcoxon signed rank test). (B) The results of intracellular IgG staining of HLA-DR^+^ PB (left) and HLA-DR^-^ PBs (right) are shown. The values represent the percentages of IgG-producing cells in each PB subpopulation. Representative data of one out of three individuals are displayed.

Previous studies showed that activated PB cells secreting IgGs would increase in the peripheral blood of healthy individuals after receiving vaccination [25,26]. To strengthen our postulate that CD138^+^HLA-DR^+^ PBs are recently activated IgG-secreting PBs, we also characterized the PB phenotypes in healthy vaccinated individuals. We found that proportions of CD138^+^ PBs, but not CD138^-^ PBs, among CD19^+^ B-cells significantly increased one week after influenza vaccination. Neither mB nor naïve B-cells (nB) were altered (Figure **3A**). This observation suggests that CD138^+^ PB cells are more enriched among germinal center-derived cells than CD138^-^ PB. Next, we compared HLA-DR^+^ and HLA-DR^-^ PBs from vaccinated individuals for the intracellular expression of IgGs. We confirmed that HLA-DR^+^ PBs were the major producer of IgGs (Figure **3B**). These results support further that CD138^+^HLA-DR^+^ PBs, which increase during the relapse of NMO, correspond to recently activated IgG-producing cells. 

### CD138^+^HLA-DR^+^ PBs upregulate CXCR3 during relapse of NMO

**Figure 4:**
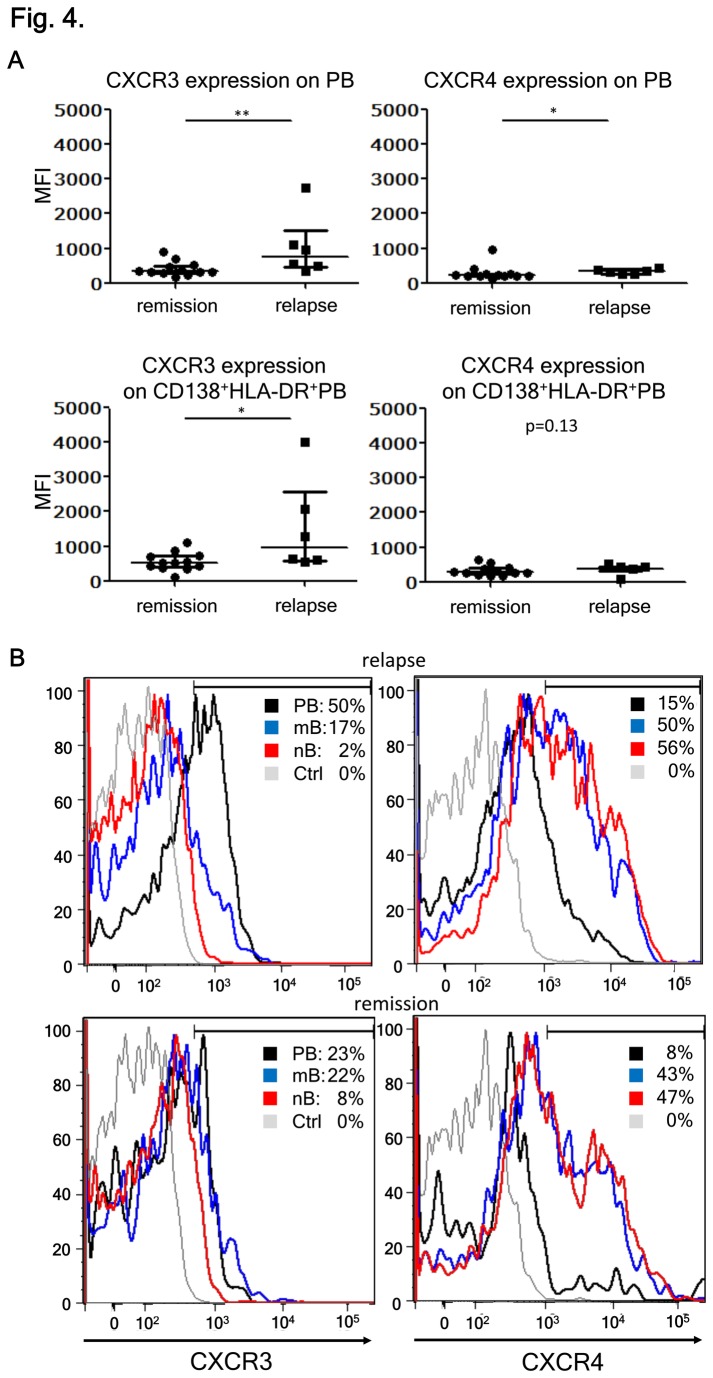
CXCR3 expression on plasmablasts (PBs) correlates with the disease state. (A) CXCR3 and CXCR4 on PB in neuromyelitis optica (NMO) relapse and remission. Here, we compared the mean fluorescence intensity (MFI) of CXCR3 and CXCR4 expression in the peripheral blood PBs during remission and relapse of NMO. MFI of CXCR3 and CXCR4 expressions on CD138^+^HLA-DR^+^ PBs were also analyzed [**p < 0.01 and *p < 0.05 by Mann–Whitney test; each error bar represents the median ± interquartile range (IQR]]. (B) B-cell subpopulations derived from peripheral blood mononuclear cells (PBMC) during disease relapse and remission were analyzed by flow cytometry to investigate the expression of CXCR3 and CXCR4. The values represent the percentages of CXCR3^+^ or CXCR4^+^ cells within each B-cell subpopulation. Unstained control of PBMC is indicated by Ctrl. Representative data of at least five patients in each disease state are shown.

Results of chemo-attractant assays showed that CXCR4 and CXCR3 ligands would attract PB cells, but not mature plasma cells [27]. The expression of CXCR4 on PB is important for their homing to the bone marrow, whereas the interaction of CXCR3 with its ligand CXCL10 plays a key role in the migration of PBs toward inflamed tissues. CXCL10 is thought to play a critical role in NMO [17]. Therefore, we analyzed the expression of CXCR4 and CXCR3 on PB cells in the peripheral blood of NMO patients. We observed that the expression levels of CXCR3 on PBs or activated PBs (CD138^+^HLA-DR^+^) were significantly higher during relapse than in remission of NMO. It appeared that the upregulation of CXCR4 on PBs during relapse was less obvious (Figure **4A**). We further analyzed the PBMC obtained during relapse and in remission for the expression of CXCR3 and CXCR4 according to B-cell subpopulations. Proportions (%) of CXCR3^+^ cells among mB and nB cells were 17% and 2%, respectively during relapse and did not differ from remission, whereas that for PBs was approximately 50% during relapse compared to 23% in remission (Figure **4B**). In contrast, the percentages of CXCR4^+^ cells were higher in mB and nB cells (50% and 56%, respectively) than that in PBs (15%) during relapse. These percentages of CXCR4^+^ B-cell subpopulations did not differ from those during remission. The increased CXCR3 expression on PBs during relapse suggests biological significance and allows us to speculate that the migration of CD138^+^HLA-DR^+^ PBs to the CNS might involve a CXCR3-dependent mechanism. 

### Peripheral PBs and CSF PB have common IgG variable regions

Single-cell sorting of lymphocytes is a refined method for characterizing the cellular properties of human B-cell lineages [10,21,25,28]. To characterize the antibody gene repertoire of the IgG-producing PBs in patients with NMO, we performed a single-cell sorting of PB clones from peripheral blood and CSF during relapse of NMO, and sequenced the gene fragments of the heavy and light chain variable regions after cloning. In principle, B-cell V regions are rearranged within the germinal centers and acquire the ability to produce high-affinity antibodies that are diversified by somatic hypermutation. We compared these variable region sequences with the germline database of IgBLAST (www.ncbi.nlm.nih.gov/igblast/) to estimate the number of mutations. In addition, the ratio of replacement to silent mutations (R/S ratio) was analyzed in single-cell sorted PBs derived from a patient with NMO. We established 38 paired vector clones containing cDNAs of heavy- and light-chain variable regions. The variable regions of the PB IgG heavy chain contained an average of 17 mutations and had higher R/S ratios in CDRs than in framework regions (FR) (Figures **S4** and **S5**). Moreover, the variable regions of the IgG κ chains of the sorted PB contained an average of 10 mutations and also had higher R/S ratios in CDRs than in FR. However, there were no significant differences in the number of mutations between the PB clones from peripheral blood and from CSF (Figure **S6**). The numbers of mutations in these PB clones did not differ from those in IgG^+^ mB [28]. These results confirmed that PB clones derived from NMO would represent a post-germinal center B-cell lineage. 

The similarity in the numbers of mutations in peripheral and CSF PB clones prompted us to address whether differentiated PBs might migrate from peripheral blood to CSF without further affinity maturation. Therefore, we compared the sequences of the CDR regions of PB clones from peripheral blood and CSF. Single-cell sorted PBs (109 from peripheral blood and 67 from CSF) were obtained from two patients with NMO in relapse. Because our target was recently differentiated PB derived from germinal centers, we selectively amplified the CDR3 regions of the IgG H-chains. The CDR3 regions of the IgG genes (41 from patient 1 and 51 from patient 2) were successfully sequenced. The clones were numbered in the order they were collected. We noticed that 11 CDR3 sequences were repeatedly detected in 17 CSF PB clones and 21 peripheral blood PB clones (Table **2**). Interestingly, five of the 11 sequences were detected in both peripheral blood and CSF clones of the same patient (clone numbers in bold), indicating the migration of the PB clones from the peripheral blood to the CSF. Interestingly, a common CDR3 sequence (VKFSATAAAGNWDHFDY) was obtained from the PB clones from both patients (peripheral blood-derived clones 28 and 30 and CSF-derived clone 36 in patient 1; peripheral clones 3 and 24 and CSF-derived clones 1, 2, and 22 in patient 2 with the CDR3 sequence). These results suggested that the PBs seem to be inclined toward several representative clones during the relapse of NMO. Although the pathological implications remain obscure, it might be important to follow up in a larger patient cohort. 

**Table 2:** V_H_ sequences of the plasmablasts (PBs) clones.

**Clones derived from peripheral blood**	**Clones derived from the cerebrospinal fluid (CSF)**	**H chain of CDR3**	**Germline**	**Joint region**
pt1-**28**, **30**	**36**	VKFSATAAAGNWDHFDY	VH3-23	J4
pt1-**26**	**12, 15**	ARGFYYGSGSRRGMDV	VH4-4	J6
pt1-22, 23	―	ARGDNGSFSY	VH3-74	J4
pt1-ー	8, 13	ARGIVTA	VH3-7	J4
pt1-ー	34, 39	ARQATEQVPVLPFVMGAPRKKGGAFNV	VH4-39	J4
pt2-33, 37, 39	―	VRDSPPPATHFDY	VH3-21	J4
pt2-**27**	**25, 30, 32, 35, 36, 40**	ARMARAGNYANNWYDP	VH4-59	J4
pt2-**3**, **24**	**1, 2, 22**	VKFSATAAAGNWDHFDY	VH3-23	J4
pt2-**4**, **6**, **10**, **12**, **13**, **20**	**8**	AREDLPGTMFDY	VH3-33	J4
pt2-23, 26	―	VRDNWGVDY	VH3-74	J4
pt2-21, 31	―	RCHRDRSGSPVGWYAP	VH4-30	J4

The IgG heavy chain sequences in the PB clones from neuromyelitis optica (NMO) patients were determined. Distinct PB clones obtained from peripheral blood and CSF samples are numbered in the order they were collected. In the table are shown the amino acid sequences of 11 CDR3 that were repeatedly detected in the PB clones, V_H_ gene (germline), and joint regions. When the same sequences were detected in both peripheral blood and CSF clones of the same patient, the clone numbers were highlighted in bold.

## Discussion

NMO is an inflammatory disease with a pathology characterized by AQP4-Ab–mediated astrocytopathy in the CNS lesions. As previously reported, IL-6 as well as IL-6-dependent PBs, a terminally differentiated B-cell population, are involved in the pathogenesis [12]. Results of laboratory works have suggested that the AQP4-Ab produced in the periphery might cause the astrocyte pathology [9–11], assuming that they could enter the CNS through the disrupted area of the BBB. However, the role of antibody production within the CNS has not been excluded in human NMO. In fact, it remains unclear why AQP4-expressing organs, including kidney and stomach, are not involved in NMO, despite the presence of AQP4-Abs in the periphery [29]. It is unknown whether PBs play a pathogenic role within the CNS after migrating to the CNS. Here, we demonstrated that CSF lymphocytes from NMO during relapse are enriched in activated PB cells, brightly expressing CD138 and HLA-DR. CD138 serves not only as a negative regulator of cell migration in vitro [23], but also as the marker of tissue migrating plasmablasts/plasma cells in vivo [27]. We have confirmed that HLA-DR expression would characterize the PB subset capable of secreting IgG in healthy vaccinated subjects (Figure **3B**). It is possible that the increased frequency of HLA-DR^+^ PBs in NMO reflects an antigen-driven B-cell activation that plays a key role in NMO pathogenicity. Moreover, we showed that the CD138^+^HLA-DR^+^ PBs would selectively upregulate CXCR3 during relapse. Because CXCL10, a CXCR3 ligand, is present in the CSF of NMO [17], we speculate that the activated PBs might migrate from the periphery to the CNS in a CXCR3-dependent manner. Intriguingly, a study on a neurotropic coronavirus-induced encephalomyelitis model showed that CXCR3-expressing PBs would infiltrate the CNS and locally produce antibodies against the pathogen [30]. The viral encephalomyelitis induced in CXCR3-knockout mice was remarkably exacerbated in association with a marked reduction of PB cells infiltration. Although this work highlights the importance of CXCR3-dependent PB migration in the production of antiviral antibodies within the CNS, it also gives us a clue to understand how migratory PBs would contribute to the pathogenesis of NMO. 

We further proved that IgG-producing PBs in the CSF during relapse share identical CDR sequences with those from PBs in the peripheral blood (Table **2**). The IgG sequences were highly mutated (Figure **S4** and **S5**), indicating that helper T-cells guided the PBs toward germinal centers. Although we did not separate CD138^−^ and CD138^+^ PBs in this analysis, the number of mutations in the H-chain variable regions showed a single-peak distribution (Figure **S4**). These results suggest that both CD138^−^ and CD138^+^ PBs are affinity-maturated B-cells, although CD138 expression levels could inversely correlate with the tissue-migrating ability [23]. There remained a possibility that mB might give rise to PBs within the CSF. However, further affinity maturation in PBs was not observed in CSF compared with peripheral blood, indicating that clonal expansion and differentiation of mB in the CSF is not a major pathway. This assumption is also supported by the rare occurrence of CSF oligoclonal bands and raised IgG index in NMO, which indicates that the intrathecal IgG synthesis was low, transient, and restricted to acute relapse in NMO patients [6,31]. Taken together, it is likely that CXCR3-expressing PBs are expanded in the periphery and recruited to the CNS in the pathogenesis of NMO. In the CNS, B-cell stimulatory cytokines such as IL-6 would support the PB survival and AQP4-Ab production, leading to the destruction of astrocytes and the glia limitans. We and another group have shown that PBs from peripheral blood and CSF produce anti-AQP4 IgG antibody in NMO [10,12]. We therefore postulated that the common IgGs shared by PBs from the PBMC and CSF (Table 2) could bind to AQP4. Despite substantial efforts, however, we have not succeeded in this attempt so far. Though we speculate that peripherally expanded PBs producing anti-AQP4 should be able to cross BBB like other PBs, irrespective of the antigen specificity, more efforts will be needed to formally prove our postulate.

We previously demonstrated the role of IL-6-dependent PBs in the production of AQP4-Ab [12]. In the present report, we indicate that PBs may play a more critical role in the CNS by locally producing AQP4-Ab. The relevance of this model can be verified in clinical trials of drugs targeting appropriate cells or molecules. In fact, we have recently shown that humanized anti–IL-6 receptor antibody (Tocilizumab) was efficacious in a patient with NMO in reducing the number of PBs in the peripheral blood as well as stabilizing the clinical conditions [32]. In another report, Tocilizumab successfully controlled three NMO patients who were resistant to the anti-CD20 antibody Rituximab [33]. These results indicate that PBs, rather than CD20^+^ mB, play a pivotal role in NMO. Therefore, it will be intriguing to test the effect of drugs altering the migration of PBs toward the CNS [34]. 

## Supporting Information

Figure S1 **Flow cytometric analysis of PB.** Flow cytometric scheme of B-cell subpopulation analysis. The partitioned cells are CD19^+^CD27^+^ cells within peripheral blood mononuclear cells (PBMC; left panel). The CD19^+^CD27^+^ cells were further analyzed to investigate the expression of CD38 and CD180 (middle panel). CD38^high^CD180^-^ cells (partitioned in the middle panel), corresponding to plasmablast (PB) cells, were analyzed again to investigate the expression of CD19 and CD27 (right panel). This result assured that the encircled population in Figure 1A represented CD19^int^CD27^high^CD38^high^CD180^-^ PB cells.
(TIF)

Figure S2 **B-cell proportions in peripheral blood mononuclear cells (PBMC) and cerebrospinal fluid (CSF) from NMO and MS.** PBMC and CSF were obtained from neuromyelitis optica (NMO) and multiple sclerosis (MS) patients. Here, we show the proportions (%) of total B-cells (CD19^+^) and CD19^+^CD27^+^ cells among the PBMC and CSF. The Mann-Whitney test provided the statistical p values. The bars represent the median ± interquartile range (IQR).
(TIF)

Figure S3 **The proportion of plasmablast (PB) cells among the total B-cells.** Peripheral blood mononuclear cells (PBMC) and cerebrospinal fluid (CSF) were obtained from neuromyelitis optica (NMO) and multiple sclerosis (MS) patients. The proportions (%) of PB cells among the total B cells (CD19^+^) are reported. The Mann-Whitney test provided the statistical p values (**p < 0.01; *p < 0.05). The bars represent the median ± interquartile range (IQR).
(TIF)

Figure S4 **The number of somatic hypermutations in plasmablast (PB) clones.** V_H_ and V_Kappa_ regions of the IgG gene were evaluated in a total of 38 PB clones derived from a patient with neuromyelitis optica NMO (Pt1) during relapse. There were 17.4 ± 1.3 [mean ± standard error of the mean (SEM)] in the V_H_ regions and 10.5 ± 1.5 mutations in the V kappa regions.
(TIF)

Figure S5 **Plasmablast (PB) cells are diversified by somatic hypermutations.** The mutation frequencies in the framework regions (FR) and in complementarity-determining regions (CDR) of the V_H_ and V_Kappa_ regions of the IgG genes were analyzed in PB clones from patient 1 (Pt1). The ratio of replacement (R, black bars) to silent (S, white bars) changes are shown at the bottom (R/S ratio).
(TIF)

Figure S6 **Comparison of the somatic hypermutations in peripheral blood mononuclear cells (PBMC)- and cerebrospinal fluid (CSF)-derived PB clones.** Here, we compare the plasmablast (PB) clones derived from the peripheral blood (N = 14) and from CSF (N = 24) with the number of mutations in the V_H_ and V_Kappa_ regions of the IgG genes. The statistic p values were obtained by Mann-Whitney test. The data represent the median ± interquartile range (IQR).
(TIF)
