# Supplementary figures and images for: Plasmablasts as Migratory IgG-Producing Cells in the Pathogenesis of Neuromyelitis Optica
Source: PLoS One. 2013 Dec 10;8(12):e83036. doi: 10.1371/journal.pone.0083036 (PMC3858367; doi:10.1371/journal.pone.0083036)

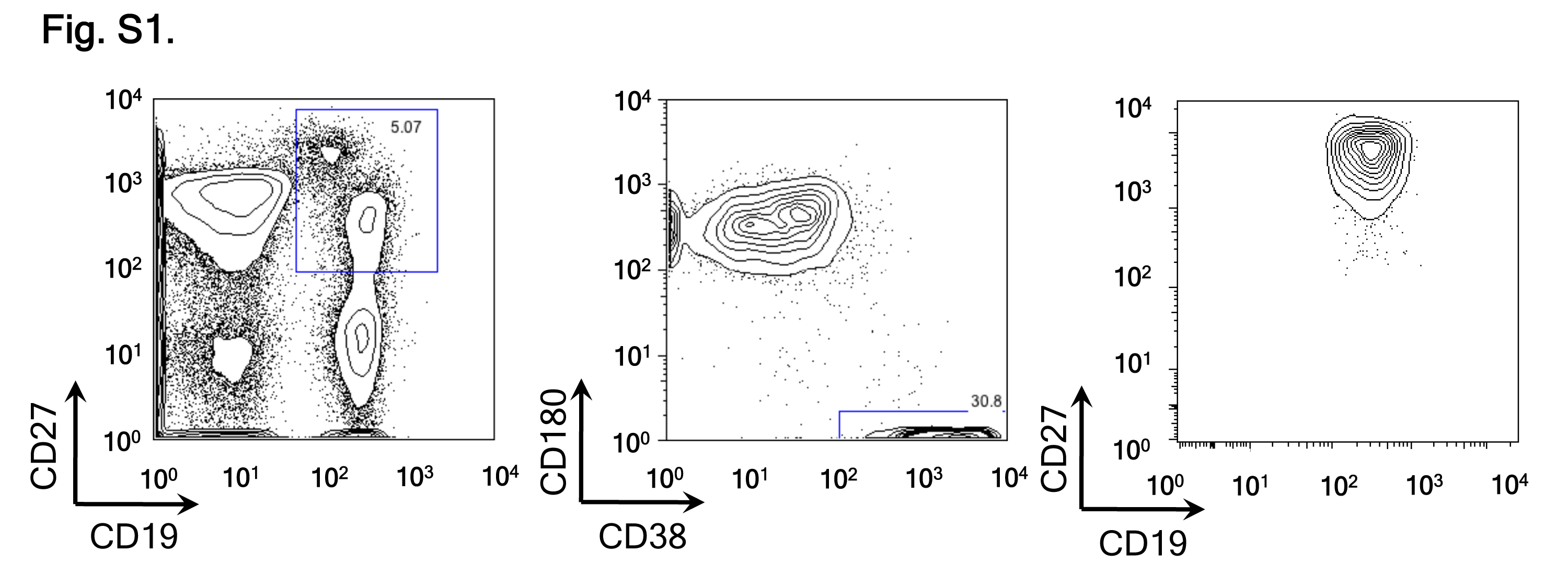

Supplement: Figure S1 — Flow cytometric analysis of PB. Flow cytometric scheme of B-cell subpopulation analysis. The partitioned cells are CD19+CD27+ cells within peripheral blood mononuclear cells (PBMC; left panel). The CD19+CD27+ cells were further analyzed to investigate the expression of CD38 and CD180 (middle panel). CD38highCD180- cells (partitioned in the middle panel), corresponding to plasmablast (PB) cells, were analyzed again to investigate the expression of CD19 and CD27 (right panel). This result assured that the encircled population in Figure 1A represented CD19intCD27highCD38highCD180- PB cells. (TIF) [file pone.0083036.s001.tif]

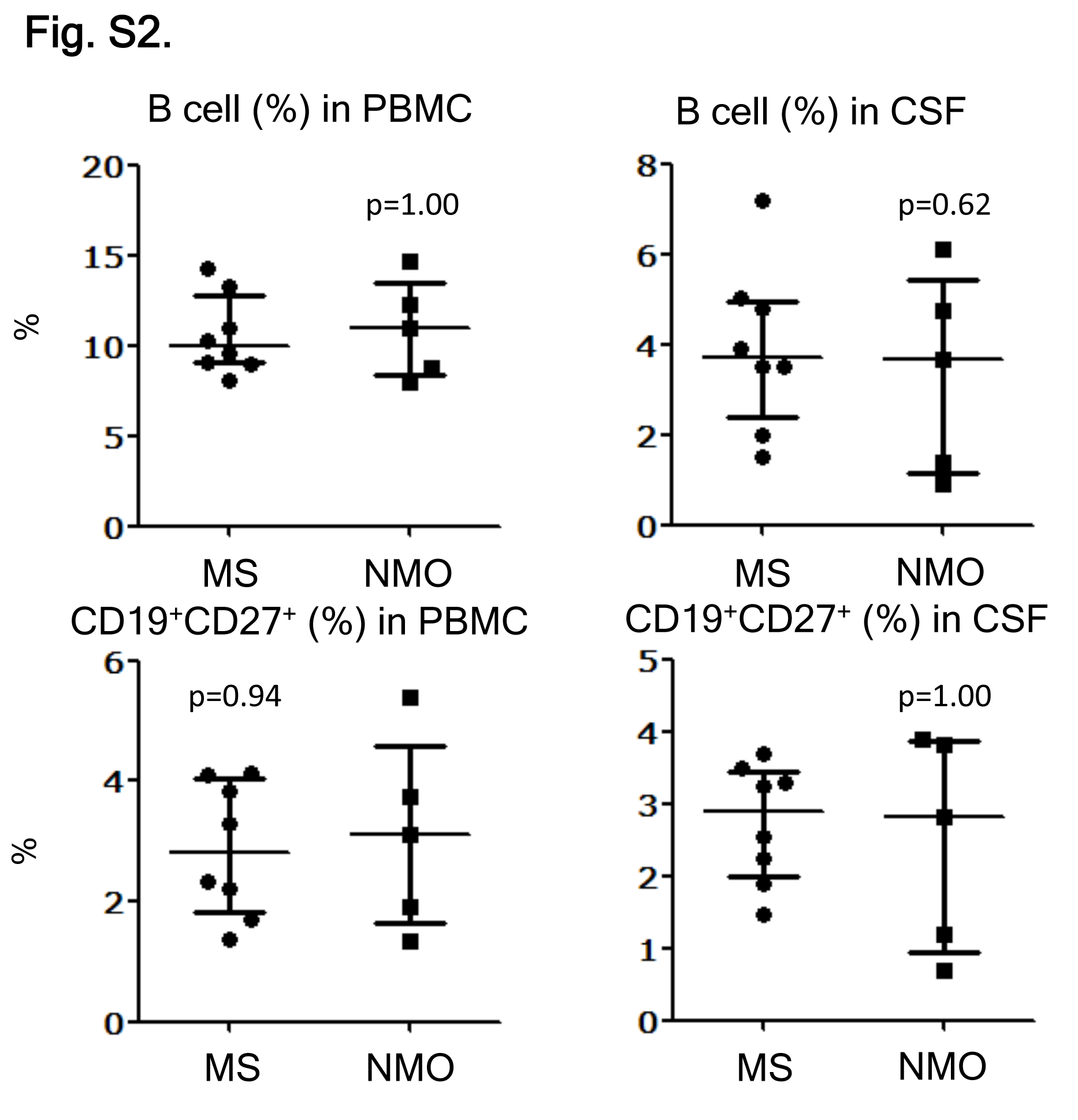

Supplement: Figure S2 — B-cell proportions in peripheral blood mononuclear cells (PBMC) and cerebrospinal fluid (CSF) from NMO and MS. PBMC and CSF were obtained from neuromyelitis optica (NMO) and multiple sclerosis (MS) patients. Here, we show the proportions (%) of total B-cells (CD19+) and CD19+CD27+ cells among the PBMC and CSF. The Mann-Whitney test provided the statistical p values. The bars represent the median ± interquartile range (IQR). (TIF) [file pone.0083036.s002.tif]

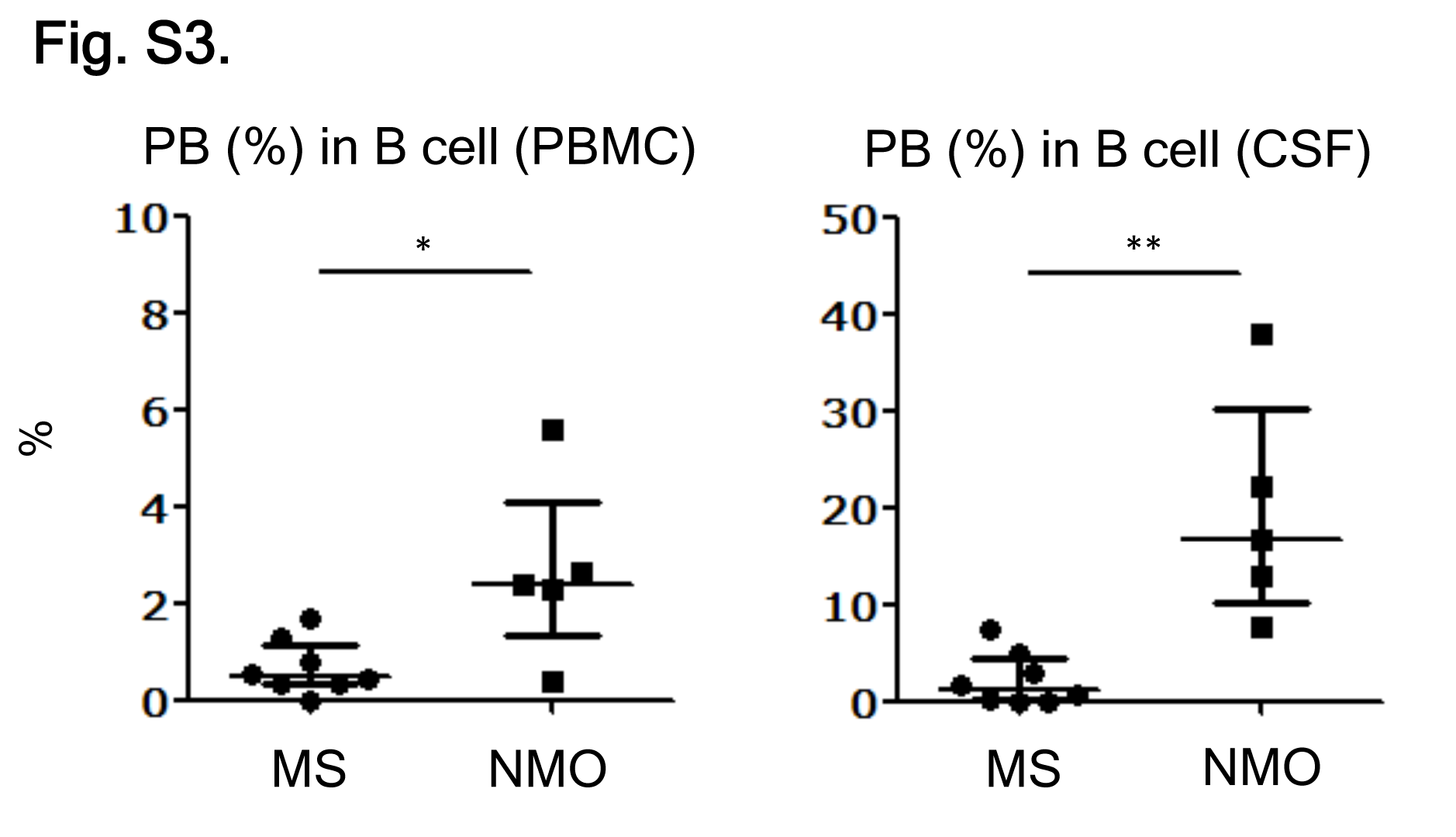

Supplement: Figure S3 — The proportion of plasmablast (PB) cells among the total B-cells. Peripheral blood mononuclear cells (PBMC) and cerebrospinal fluid (CSF) were obtained from neuromyelitis optica (NMO) and multiple sclerosis (MS) patients. The proportions (%) of PB cells among the total B cells (CD19+) are reported. The Mann-Whitney test provided the statistical p values (**p < 0.01; *p < 0.05). The bars represent the median ± interquartile range (IQR). (TIF) [file pone.0083036.s003.tif]

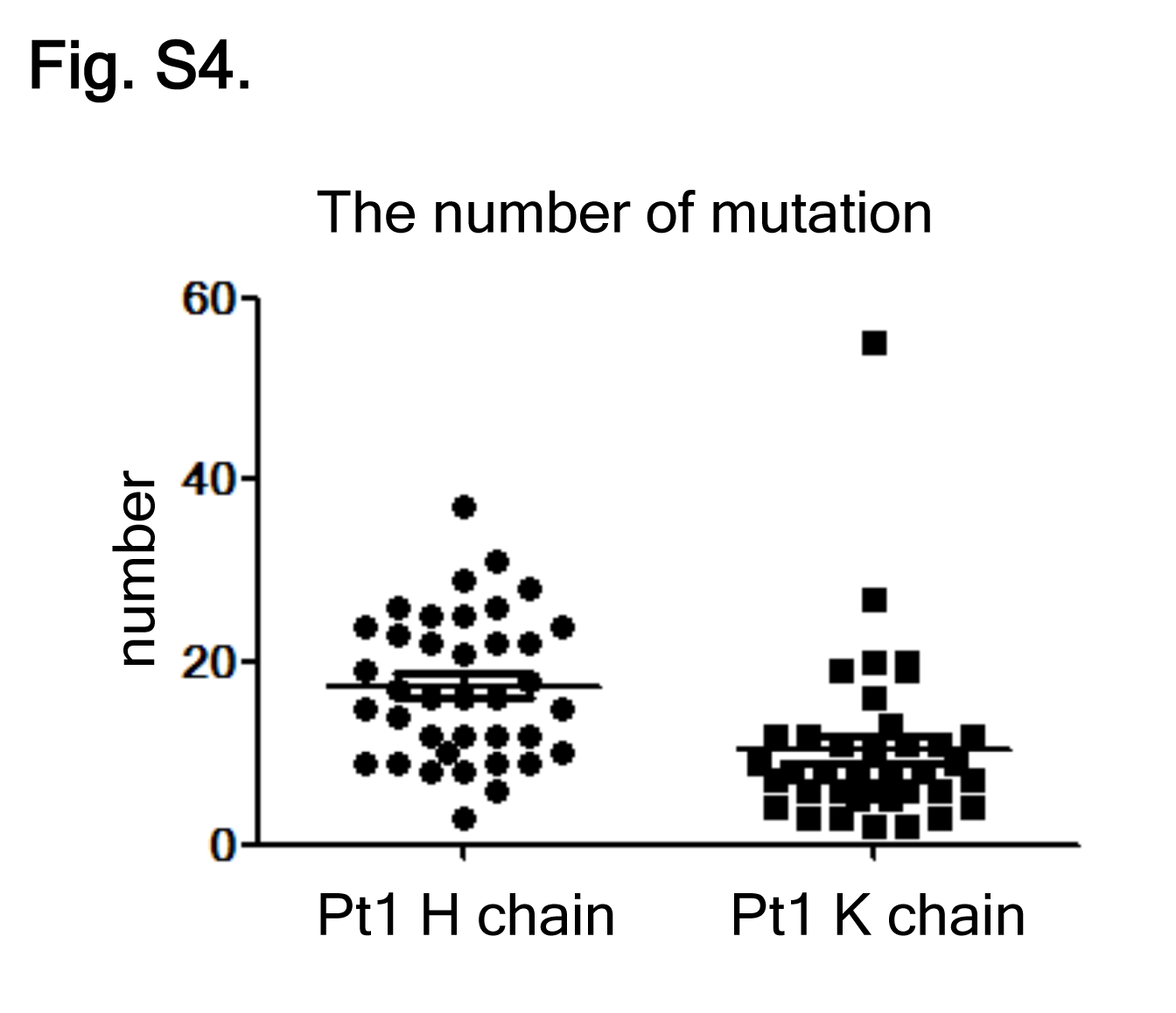

Supplement: Figure S4 — The number of somatic hypermutations in plasmablast (PB) clones. VH and VKappa regions of the IgG gene were evaluated in a total of 38 PB clones derived from a patient with neuromyelitis optica NMO (Pt1) during relapse. There were 17.4 ± 1.3 [mean ± standard error of the mean (SEM)] in the VH regions and 10.5 ± 1.5 mutations in the V kappa regions. (TIF) [file pone.0083036.s004.tif]

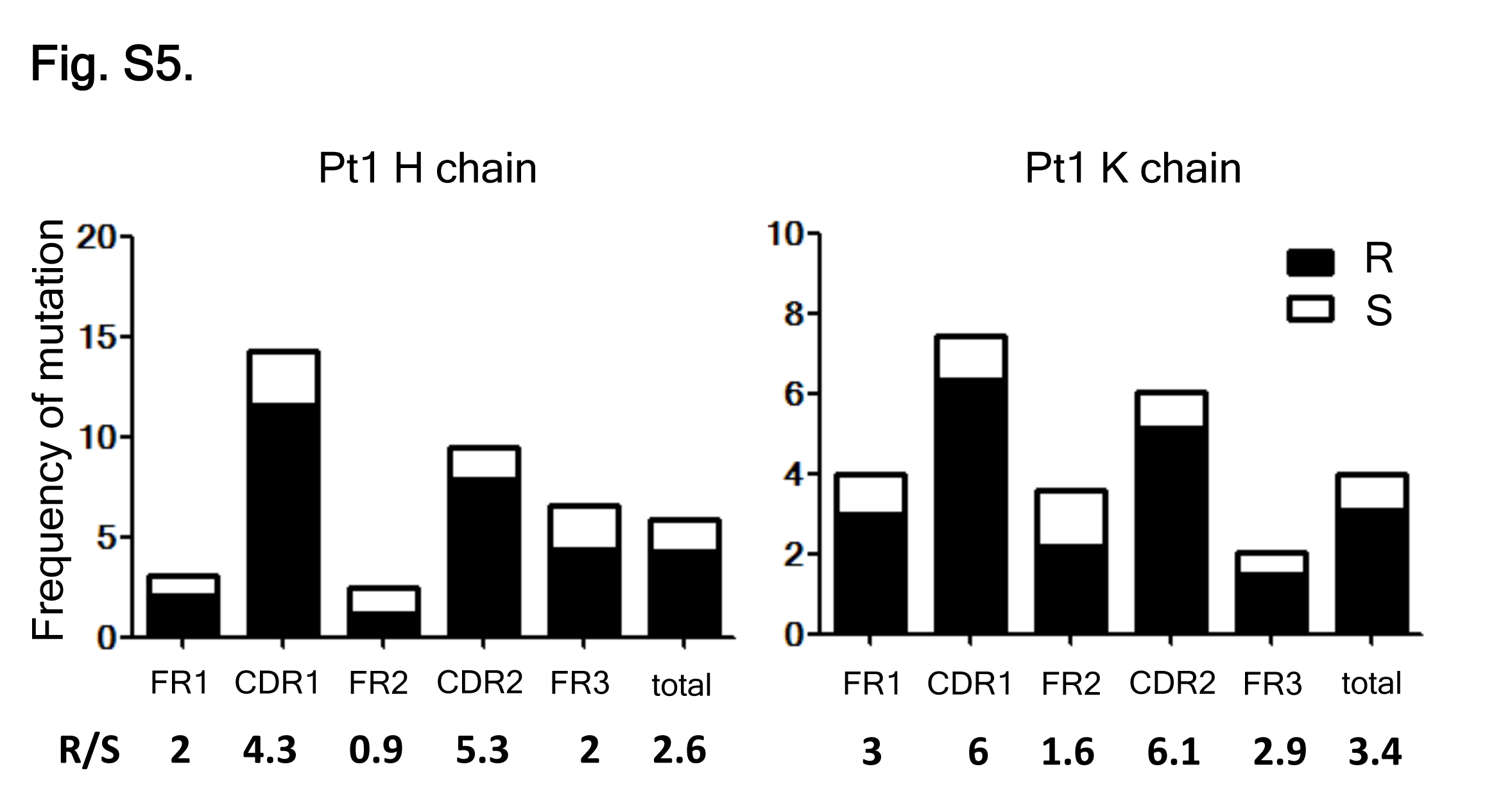

Supplement: Figure S5 — Plasmablast (PB) cells are diversified by somatic hypermutations. The mutation frequencies in the framework regions (FR) and in complementarity-determining regions (CDR) of the VH and VKappa regions of the IgG genes were analyzed in PB clones from patient 1 (Pt1). The ratio of replacement (R, black bars) to silent (S, white bars) changes are shown at the bottom (R/S ratio). (TIF) [file pone.0083036.s005.tif]

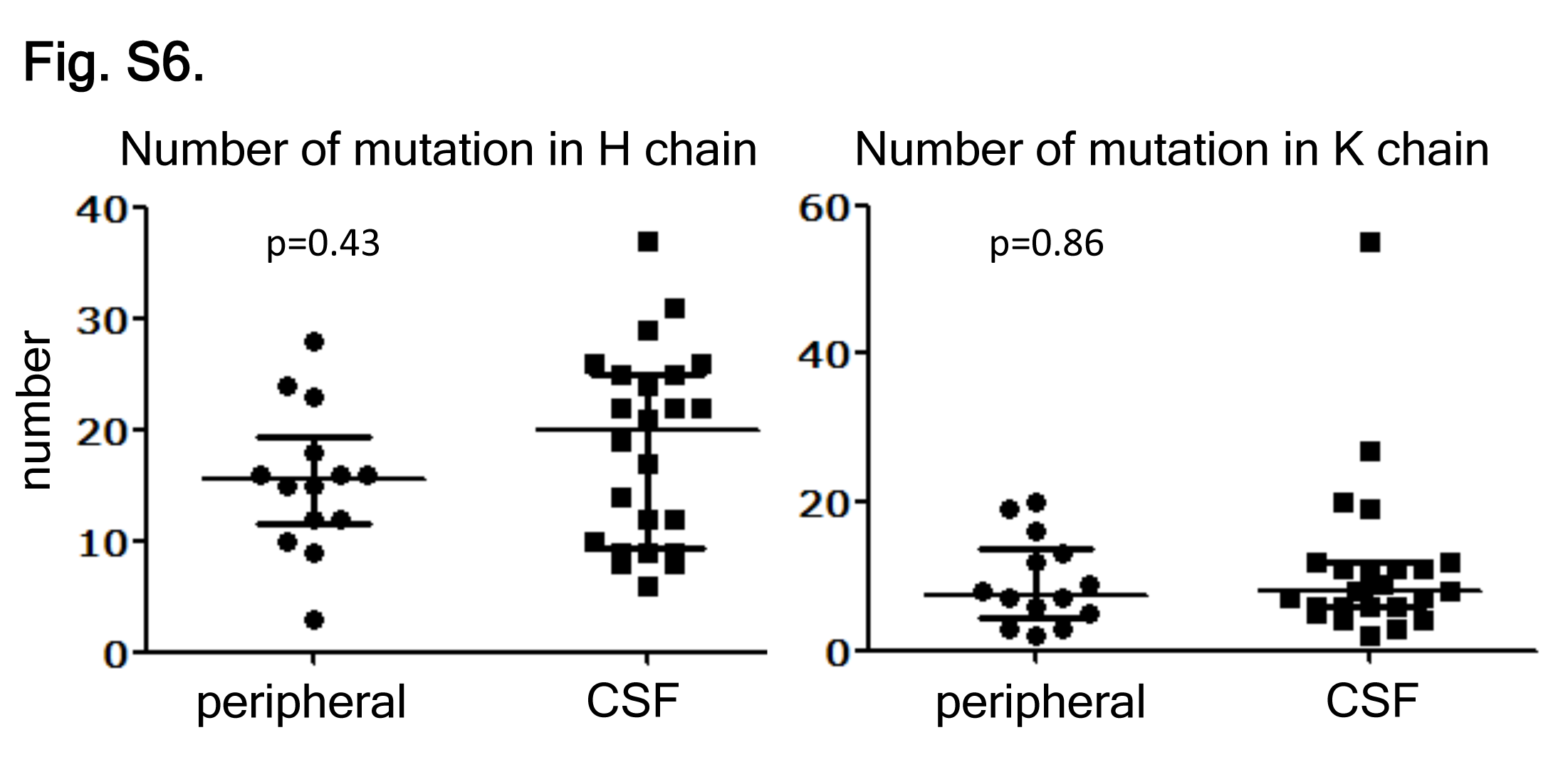

Supplement: Figure S6 — Comparison of the somatic hypermutations in peripheral blood mononuclear cells (PBMC)- and cerebrospinal fluid (CSF)-derived PB clones. Here, we compare the plasmablast (PB) clones derived from the peripheral blood (N = 14) and from CSF (N = 24) with the number of mutations in the VH and VKappa regions of the IgG genes. The statistic p values were obtained by Mann-Whitney test. The data represent the median ± interquartile range (IQR). (TIF) [file pone.0083036.s006.tif]
